# Change in smoking cessation stage over 1 year in patients with schizophrenia: a follow up study in Japan

**DOI:** 10.1186/s12888-019-2351-9

**Published:** 2019-11-21

**Authors:** Yuji Higuchi, Masaki Fujiwara, Naoki Nakaya, Maiko Fujimori, Chinatsu Hayashibara, Ryuhei So, Ikuta Shinkawa, Kojiro Sato, Yuji Yada, Masafumi Kodama, Hiroshi Takenaka, Yoshiki Kishi, Kyoko Kakeda, Yosuke Uchitomi, Norihito Yamada, Masatoshi Inagaki

**Affiliations:** 1 Taiyo Hills Hospital, 2200 Abe, Ochiaicho, Takahashi City, Okayama, 716-0061 Japan; 2 Department of Neuropsychiatry, Okayama University Hospital, 2-5-1 Shikata-cho, Kita-ku, Okayama, 700-8558 Japan; 3 Department of Preventive Medicine and Epidemiology, Tohoku Medical Megabank Organization, Tohoku University, 2-1 Seiryo, Sendai, 980-8573 Japan; 4 Division of Health Care Research, Behavioral Sciences and Survivorship Research and Division of Cohort Consortium Research, Epidemiology and Prevention Group, Center for Public Health Sciences, National Cancer Center, Tsukiji, Chuo-ku, Tokyo, 104-0045 Japan; 5 Division of Occupational Therapy, Faculty of Care and Rehabilitation, Seijoh University, 2-172 Fukinodai, Tokai City, Aichi 476-8588 Japan; 6 Okayama Psychiatric Medical Center, 3-16 Shikatahon-machi, Kita-ku, Okayama, 700-0915 Japan; 7 Department of Neuropsychiatry, Kochi Medical School, Kochi University, Kohasu, Oko-cho, Nankoku, Kochi 783-8505 Japan; 8 Innovation Center for Supportive, Palliative and Psychosocial Care, National Cancer Center Hospital and Behavioral Sciences and Survivorship Research, Center for Public Health Sciences, National Cancer Center, Tsukiji, Chuo-ku, Tokyo, 104-0045 Japan; 9 Department of Neuropsychiatry, Okayama University Graduate School of Medicine, Dentistry, and Pharmaceutical Science, 2-5-1 Shikata-cho, Kita-ku, Okayama, 700-8558 Japan; 10 Department of Psychiatry, Faculty of Medicine, Shimane University, 89-1 Enya-cho, Izumo, Shimane 693-8501 Japan

**Keywords:** Cigarettes, Mental health, Schizophrenia, Smoking, Tobacco products

## Abstract

**Background:**

We performed a follow up study about willingness and behaviors to quit smoking among smokers with schizophrenia in Japan.

**Methods:**

Participants were outpatients with schizophrenia aged 20–69 years who had been visiting the hospital for ≥1 year as of April 1, 2016, and had visited the hospital more than once in the previous 6 months. A baseline survey on smoking behaviors including current smoking status and smoking cessation stage, was administered in 420 participants that were randomly extracted from a patient pool (*n* = 680) in 2016, and a follow-up survey was administered in 2017. We calculated the distribution and change in smoking cessation stage, number of smokers and nonsmokers after 1 year, and quitting rate from a naturalistic 1-year smoking-cessation follow up.

**Results:**

The number of baseline respondents was 350; 113 current smokers and 68 former smokers. Among the 113 current smokers, 104 (92.0%) were followed for 1 year, 79 (70.0%) were interested in smoking cessation, and only 7 had received smoking cessation treatments at baseline. Among the tracked 104 participants, only 6 (5.8%) stopped smoking after 1 year. Among the 25 participants who had intentions to quit smoking within 6 months at baseline, 6 (24.0%) maintained their intention to quit smoking for 1 year, and 16 (64.0%) did not maintain their intention to quit smoking.

**Conclusions:**

Our findings showed that many smokers with schizophrenia were interested in quitting smoking, but few patients received treatment and actually quit smoking. Timely intervention, including the option to receive smoking cessation treatment, is necessary for those patients with schizophrenia who smoke.

**Trial registration:**

UMIN Clinical Trials Registry (UMIN000023874, registered on August 31, 2016).

## Background

The debate over the relationship between tobacco and schizophrenia has been ongoing for some decades [1–3]. Current smoking prevalence is particularly high among those with schizophrenia, compared with the general population [4, 5]. The high prevalence of smoking in people with severe mental illness contributes substantially to their premature death because of ischemic heart disease, cancer, respiratory illnesses [6] or suicidality [7]. Therefore, we must urgently develop effective interventions to help people with schizophrenia quit smoking [8]. Unfortunately, smoking cessation rates among people with mental illness are significantly lower than among those without mental illness [9], despite the fact that most countries have achieved significant annualized rates of decline in smoking prevalence in their general populations [10]. The unchanged high smoking rate among people with mental illness may be due in part to disparities in support schemes for tobacco-use reduction [11].

Older theories have considered smoking in patients with schizophrenia to be inevitable, as follows: “Tobacco is self-medication for schizophrenia patients because nicotine relieves their symptoms. Therefore, they would not quit smoking, and smoking cessation may exacerbate their illness.” However, more recent evidence has failed to support these claims, and has shown that patients with schizophrenia can quit smoking without threatening their mental health recovery [11–13]. Smoking cessation should be an integral part of serious mental illness treatment [14].

Social and cultural contexts influence smoking behavior [15]. However, it is unclear whether findings from Western countries are applicable to Asian countries, because the latter have relatively higher smoking rates, and the smoking rate for women is much lower than that for men [16]. Therefore, it is necessary to research the associations unique to each country between patients’ mental-health backgrounds and smoking behavior to support their smoking cessation. Although we reported previously, using anonymized national data, that serious psychological distress increases smoking behavior [17], studies in patients with schizophrenia are limited. Some previous studies have targeted hospitalized patients, but it is difficult to apply these findings to outpatients, who make up the majority of patients with schizophrenia.

Smoking cessation treatment was approved in Japan by governmental health insurance in 2006; however, basic information regarding smoking behavior in patients with schizophrenia is unknown, including whether they intend to quit smoking and how they quit smoking. Although smoking rates in patients with schizophrenia are expected to be high, we assumed that this rate is not attributable specifically to schizophrenia, but instead, to the lack of support for smoking cessation. In a well-known report in 1918 about private confinement at home, Kure and Kashida described the disparity in health care that patients with schizophrenia were experiencing at that time in Japan as follows: “Hundreds of thousands of mental patients in our country not only have to suffer the misfortune that brought this illness, but they also have to suffer the misfortune of being born in this country.” [18] One-hundred years after this report, the discriminatory environment surrounding patients with schizophrenia in Japan might not have changed. For example, smoking is still allowed in many psychiatric hospitals, although approximately 70% of the hospitals provide information or instructions about smoking cessation support for patients [19]. Thus, we performed an observational survey named as the Study of Health Behavior in People with Schizophrenia (SHEAPS), to gain information on unavoidable disadvantages related to health behavior experienced by these patients, regardless of the nature of the disease.

The purpose of the present survey was to improve the quality of smoking cessation support for smokers with schizophrenia in Japan. Thus, we performed a naturalistic 1-year follow up study about schizophrenia outpatients’ smoking behavior and change in smoking behavior according to sex (how many of each sex smoke) and to what extent they are willing to quit smoking, whether or not they had tried to quit smoking, and how they quit.

## Methods

### Study design, ethical approval, and setting

The SHEAPS has two investigation aims, as follows: (I) cancer-screening behaviors [20]; and (II) smoking behaviors in patients with schizophrenia (the present study).

SHEAPS was registered in a clinical trial registry (UMIN000023874), and was approved by the institutional ethics committee of the Okayama University Graduate School of Medicine, Dentistry, and Pharmaceutical Sciences and Okayama University Hospital (approval number KEN1608–010), and by the institutional review board of Okayama Psychiatric Medical Center (approval number 27–38). Potential participants in SHEAPS were informed that they could decline to participate or withdraw from the study at any time, and all participants provided written informed consent. Participants were recruited in a psychiatric outpatient clinic at the Okayama Psychiatric Medical Center in Japan. This hospital, which has 252 beds and approximately 250 outpatient visits per day, is the core public psychiatric hospital in the area and cares for a wide range of patients, from chronic outpatients to acute and severe inpatients. SHEAPS-(II) was performed from 2016 to 2017, and, to the best of our knowledge, is the first follow-up report investigating schizophrenia outpatients’ smoking behavior in Asia.

### Participants

As described previously [20], the SHEAPS inclusion criteria were as follows: On 1 April 2016, patients: (i) were aged 20–69 years; (ii) had visited the hospital for ≥1 year and visited two or more times in the previous 6 months; and (iii) in July 2016 (eligibility assessment time), had been diagnosed with schizophrenia or a schizoaffective disorder (but not other schizophrenia spectrum disorders) according to the fifth edition of the Diagnostic and Statistical Manual of Mental Disorders, by their primary psychiatrist [21]. The exclusion criteria were as follows: (i) patients comorbid for intellectual disabilities and unable to complete the questionnaires; (ii) those with psychiatric or physical symptoms too severe to make study participation appropriate (as judged by their primary psychiatrist); and (iii) those illiterate in Japanese. In accordance with the criteria, 680 patients were pooled as eligible participants from the hospital records and judgments by their primary psychiatrist. We estimated that the number of patients we could contact within 3 months would be 420, based on time- and human resources. These 420 patients were randomly sampled from the patient pool (*n* = 680) in August 2016 using computer-generated random numbers by a researcher blinded to the other information except for the study identification number. We asked the extracted patients to participate in SHEAPS from September to November 2016. The authors contacted most participants directly at the hospital, and also attempted to increase participation by approaching other potential participants via telephone and mail. We tracked participants defined as smokers in our 2016 baseline survey, and asked them to participate again in the follow-up survey from September to November 2017. We did not follow potential smokers who did not smoke at baseline; therefore, we missed some who might have started smoking between the surveys.

### Data source and measurements

Sex and age data were obtained from participants’ medical records. Educational level and marital status were self-reported on a paper-based questionnaire. The researchers and a trained research assistant helped participants complete the questionnaires, as needed. In both the 2016 and 2017 surveys, participants were asked to answer questions about smoking status, smoking cessation stage, and the methods of smoking cessation.

### Smoking status

Participants were asked to disclose their smoking status to identify “current smoker”. According to World Health Organization guidelines [22], a participant is defined as “a current smoker” if he/she responds “yes” to all the three questions, as follows: “Do you currently smoke?”, “Have you smoked at least 100 cigarettes in your entire life or smoked for over 6 months so far?”, and “Have you smoked during the 30 days preceding the survey?”

### Smoking cessation stage

For smoking cessation status, participants were asked to select from the following four categories: stage 1 (the immotive stage): “I am not interested in smoking cessation.”; stage 2 (the precontemplation stage): “I am interested in smoking cessation, but have no plan within the next 6 months.”; stage 3 (the contemplation stage): “I’d like to quit smoking within the next 6 months, but I have no plan within 1 month.”; and stage 4 (the preparation stage): “I’d like to quit smoking within 1 month.” [23] These categories are based on the transtheoretical model and stages of change theory [24, 25], which posits that individuals progress through these four stages of change on their way toward adopting a healthy behavior or toward cessation of an unhealthy behavior before making and sustaining the behavior change. Thus, smokers in the four stages differ from each other, and would have different outcomes related to quitting tobacco in the future [26].

### Methods of smoking cessation

Participants were also asked to answer a question to confirm cessation-aids use [27], as follows: “Did you quit smoking for 24 hours or more aiming to quit smoking in the past year?” When they responded “yes” to this question, they were also asked to answer the next question about the methods of smoking cessation and to select from the following options: 1) smoking cessation treatment at a hospital, or 2) other methods including self-directed methods.

### The heavy smoking index

The Heavy Smoking Index (HSI) is composed of two items derived from the Fagerström Test for Nicotine Dependence [28, 29]. Participants were asked to answer two questions about the mean number of cigarettes smoked per day and the time from waking to smoking the first cigarette. This test is used to estimate the degree of nicotine dependence among daily smokers [30], and consists of two items with an overall score of 0–6 with higher HSI scores indicating greater nicotine dependence.

### Outcomes

The primary outcomes of this study were as follows: 1) the percentage of participants at each smoking cessation stage, and 2) change in smoking cessation stage after 1 year among current smokers in the cohort. Secondary outcomes were as follows: 3) smoking cessation rate, and 4) methods of smoking cessation. These outcomes were reported by sex.

## Results

**Fig. 1:**
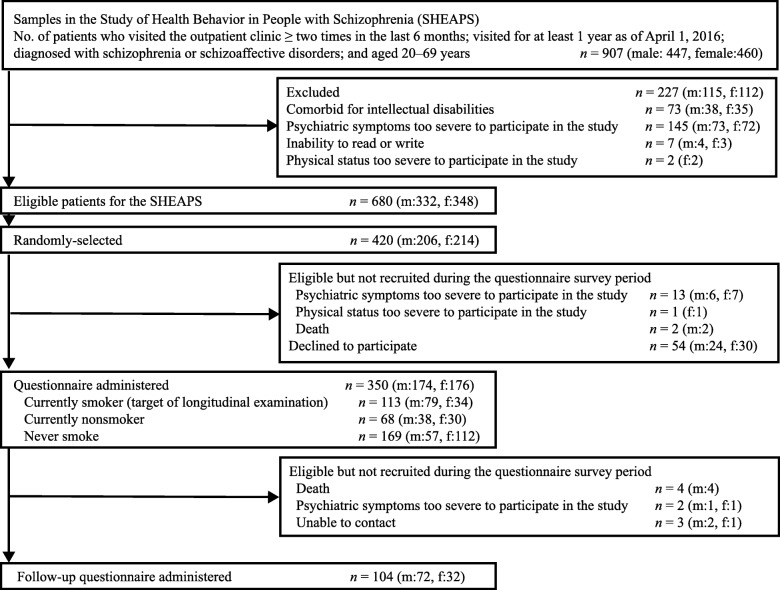
Flow diagram of the participant recruitment process. m, male; f, female

Fig. 1 shows the study recruitment process. Data were analyzed for 350 participants (83.3%) of the 420 patients randomly extracted from the 680-patient pool, and 70 were eligible but not recruited during the survey period because of their medical condition or decline. Of the 350 participants, 113 (32.2%) were current smokers, 68 (19.4%) were current nonsmokers (former smokers), and 169 (48.3%) had never smoked. Thus, the follow up study was scheduled for the 113 current smokers, and 104 participants (tracking rate, 92%) were successfully tracked after 1 year.

Tables 1 and 2 show participants’ characteristics and the current smokers’ HSI scores by sex. The number of current nonsmokers who had already quit smoking at baseline included 38 (21.8%) men and 30 (17.0%) women, and among these, 4 (10.5%) men and 3 (10.0%) women had received smoking cessation treatment. Among the 79 male and 34 female participants who were still smoking at baseline, 39 (49.3%) men and 17 (50.0%) women had failed in their smoking cessation attempts in the year before baseline, and 6 (15.4%) of the men and 1 (5.8%) woman had received smoking cessation treatment.

**Table 1:** Male participants' characteristics (n=174)

	Current smoker	Current nonsmoker	Never smoker
n (%)	79 (45.4%)	38 (21.8%)	57 (32.7%)
Age (y) mean ± SD [min-max]	45.6 ± 11.1 [22–69]	49.8 ± 11.3 [27–66]	39.9 ± 11.2 [20–67]
CP mean ± SD [min-max]^a^	693 ± 431 [38–2011]	545 ± 359 [25–1645]	680 ± 490 [0–2556]
mGAF score mean ± SD [min-max]^b^	53 ± 15 [21–88]	51 ± 13 [25–82]	51 ± 13 [21–81]
Marital status			
married	9	14	3
unmarried	58	23	50
separated	1	0	1
divorced	11	1	3
widowed	0	0	0
Educational level			
≤ junior high school	15	6	9
> junior high school but ≤high school			
	38	12	26
> high school but ≤junior/vocational college			
	12	8	7
≥ university or college	14	12	15
Heavy Smoking Index score			
0	4	–	–
1	4	–	–
2	8	–	–
3	20	–	–
4	22	–	–
5	12	–	–
6	9	–	–
Smoking cessation trial			
failed	39	–	–
no	40	–	–
Method of smoking cessation			
treatment	6	4	–
self-directed	33	34	–

^a^Equivalent dose of chlorpromazine (CP)

^b^The modified Global Assessment of Functioning scale (mGAF)

*SD* standard deviation

**Table 2:** Female participants' characteristics (n=176)

	Current smoker	Current nonsmoker	Never smoker
n (%)	34 (19.3%)	30 (17.0%)	112 (63.6%)
Age (y) mean ± SD[min-max]	41.3 ± 10.8 [22–60]	46.4 ± 10.4 [24–63]	46.0 ± 13.3 [21–69]
CP mean ± SD [min-max]^a^	694 ± 464 [75–1886]	501 ± 287 [71–1212]	509 ± 380 [0–1767]
mGAF mean ± SD score [min-max]^b^	53 ± 15 [24–88]	51 ± 14 [25–85]	56 ± 15 [20–86] (*n* = 5: missing)
Marital status			
married	8	9	39
unmarried	20	8	55
separated	0	2	1
divorced	6	9	12
widowed	0	2	5
Educational level			
≤ junior high school	5	1	11
> junior high school but ≤high school			
	15	14	50
> high school but ≤junior/vocational college			
	9	13	34
≥ university or college	5	2	17
Heavy Smoking Index score			
0	2	–	–
1	2	–	–
2	4	–	–
3	6	–	–
4	13	–	–
5	3	–	–
6	4	–	–
Smoking cessation trial			
failed	17	–	–
no	17	–	–
Method of smoking cessation			
treatment	1	3	–
self-directed	16	27	–

^a^Equivalent dose of chlorpromazine (CP)

^b^The modified Global Assessment of Functioning scale (mGAF)

*SD* standard deviation

Table 3 shows the numbers of participants at each smoking cessation stage for current smokers at baseline and at follow-up for outcome 1), and the number of those who quit smoking during the surveys for outcome 3). Smokers at smoking cessation stage ≥2 (interested in smoking cessation) included 51 (64.6%) men and 28 (82.3%) women at baseline. At the end of the follow-up survey in 2017, the numbers of smokers at stage ≥2 included 46 (69.9%) men and 24 (81.2%) women, and only 4 (5.6%) men and 2 (6.3%) women successfully quit smoking.

**Table 3:** Smoking cessation stage of current smokers in 2016 and 2017 by sex

		Current smoker in 2016		Current smoker in 2017	
		men *n* = 79 (100%)	women *n* = 34 (100%)	men *n* = 72 (100%)	women *n* = 32 (100%)
Smoking cessation stage	stage 1	28 (35.4%)	6 (17.7%)	22 (30.1%)	6 (18.8%)
	stage 2	33 (41.8%)	18 (53.0%)	40 (55.6%)	18 (56.2%)
	stage 3	9 (11.4%)	6 (17.7%)	3 (4.2%)	3 (9.4%)
	stage 4	9 (11.4%)	4 (11.8%)	3 (4.2%)	3 (9.4%)
Stopped smoking		–	–	4 (5.6%)	2 (6.3%)

**Fig. 2:**
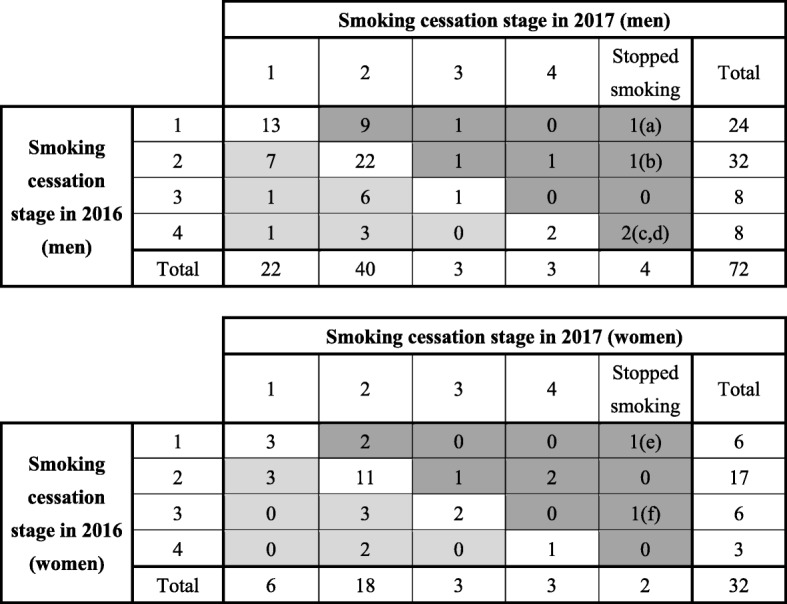
Changes in smoking cessation stage from 2016 to 2017 by sex and age, method of smoking cessation, and HSI. (a) (male) 67 y, self-directed, HSI = 2; (b) (male) 43 y, self-directed, HSI = 3; (c) (male) 63 y, self-directed, HSI = 0; (d) (male) 42 y, self-directed, HSI = 0; (e) (female) 46 y, hospital-directed treatment, HSI = 1; (f) 58 y, self-directed, HSI = 4. HSI, Heavy Smoking Index. Stage 1 (the immotive stage): “I am not interested in smoking cessation.”. Stage 2 (the precontemplation stage): “I am interested in smoking cessation, but have no plan within the next 6 months.”. Stage 3 (the contemplation stage): “I’d like to quit smoking within the next 6 months, but I have no plan to within 1 month.”. Stage 4 (the preparation stage): “I’d like to quit smoking within 1 month”

Fig. 2 shows how the smoking cessation stage changed between surveys by sex for outcome 2 and the details of participants who successfully stopped smoking between surveys for outcome 4. The highest number of participants were at stage 2 at both baseline and follow-up. According to the transtheoretical theory, after a 1-year follow-up, all participants at stage ≥3 should have stopped smoking. However, only 6 participants quit smoking, and 16 participants were at an earlier stage compared with baseline. In men, among six smokers at stage 4 at baseline, two quit smoking on their own. No women quit smoking among the three women at stage 4; however, among five women at stage 3 at baseline, one quit smoking on her own. Among the six men and women who quit smoking, only one received smoking cessation treatment, and the remaining five quit on their own. Participants at stage ≥2 at both baseline and follow-up included 36 (50.0%) men, and 22 (68.8%) women.

## Discussion

As reference data, the smoking rate we independently calculated from data from the Okayama City Comprehensive Survey of Living Conditions 2013 provided by the Ministry of Health, Labour and Welfare in Japan was 35.4% for men and 10.0% for women [31]. We used these data because approximately 60% of our participants lived in the same area, and the most recent available data on smoking rates for external reference were reported in the 2013 Ministry of Health, Labour and Welfare national survey. The smoking rate in the present study was 45.4% for men and 19.3% for women. This result is similar to the latest meta-analysis from Japan reporting smoking rates among patients with schizophrenia (men: 52.9%, women 24.4%) [5]. Thus, the smoking rate in patients with schizophrenia in the present study was higher than the reference for the general population for approximately the same time and area, as we hypothesized. Our results showed that more than half of the patients with schizophrenia in our study who had smoked were interested in smoking cessation, but results also showed that < 10% of smokers were able to achieve smoking cessation in 1 year. By tracking the stage changes in this study, we saw that some participants who had intentions to quit smoking within at least 6 months from baseline, retreated from the contemplation/preparation stage to the immotive/precontemplation stage (not interested in/interested in but not within 6 months). Many participants stayed at the precontemplation stage and did not advance.

A previous long-term follow up study reported smoking reduction among smokers with schizophrenia from the results of smoking status change [32]. In the current survey about smoking cessation stage, many participants dropped in stage, which confirms that it is difficult to achieve smoking cessation. A previous study reported that patients with schizophrenia do want to quit, but typically do not quit in the absence of evidence-based smoking cessation treatment, such as pharmacotherapy with behavioral counseling [33]. Complex active strategies that include evidence-based interventions and environmental adjustment, such as smoke-free hospital settings both indoors and outdoors and prohibition of staff smoking, should be implemented regardless of the individual’s smoking cessation stage. However, it should be noted that individuals’ attitudes, strategies, and skills differ at varying stages of the health-behavior change process [34], and these stages reflect the readiness of behavior change. Although there is limited evidence to support the efficacy of stage-based interventions in changing smoking behavior [35], we might need to intervene for patients with schizophrenia who want to quit smoking, as the present study shows. However, the type of instructive intervention needed at each smoking stage to help patients progress in the smoking cessation stages has not yet been established.

In the Japanese general population, the annual rate of smoking cessation attempts is 28.3%, and the annual rate of specialized smoking cessation treatment at hospitals is 7.4% [36]. We found that the selection ratio of smoking cessation treatment among those who tried to quit smoking in this study was comparable, though many failed, and at baseline, most women did not choose smoking cessation aids such as treatment at hospitals. There may be insufficient opportunities for smoking cessation treatment and education, especially for women with schizophrenia. Smoking cessation drugs are reportedly effective and safe in assisting smoking cessation in patients with schizophrenia [37], and these medications might be easily obtained to begin immediate treatment [38]. One meta-analysis showed that the cessation rates after treatment of smoking cessation drugs were significantly higher than placebo treatment (risk ratio: 3.03–4.74) [39]. Valenicline has been reported to be more effective than nicotine patches in women [40]. However, a web-based survey in Japan reported that over 70% of smokers did not use any therapy or assistance [41], and national data have shown that many psychiatric institutions do not offer smoking cessation treatment by public health insurance in Japan [42]. Therefore, the importance of drug treatment needs to be recognized.

This study has several limitations that should be considered. First, the self-reported data could have resulted in misclassification of data. However, self-reported smoking status is reported to be positively-related to the carbon monoxide level in expired air, and this is a reliable method for evaluating the smoking status of patients with schizophrenia regardless of their sex [43, 44]. Second, participants were recruited from a single outpatient clinic in a psychiatric hospital. Thus, the generalizability of data from this population has a clear limit. For this reason, we did not analyze the associations between patients’ background characteristics and smoking behavior, and focused mainly on changes in smoking preference. Second, our sample included many severely-affected patients, and generalization of the results is limited because of selection bias. Patients with more severe symptoms might have been more likely to decline to participate in this survey or to answer the questionnaire, which could have led to an underestimation of the number of current smokers. Third, the applicability of our findings to other countries with different health care systems regarding smoking cessation is unknown. Fourth, we only surveyed the intention to quit smoking, and did not investigate how much the number of cigarettes per day changed. Therefore, we cannot make any conclusions about the relationship between stage change and behavior change. Fifth, to date, there is little research on changes in smoking cessation levels in the general Japanese population that can be compared with this report. Therefore, future reports about this issue are needed.

## Conclusion

This study showed that few patients were offered aids and few patients quit smoking, although many smokers with schizophrenia were interested in smoking cessation. Without smoking cessation aids, intentions for smoking cessation may not proceed or may decrease. Considering that only 10% of our participants received smoking cessation treatment (even though many intended to quit smoking), interventions for cessation treatment are necessary, especially for those at the contemplation/preparation stage. It is necessary to develop effective smoking cessation interventions for smoking patients with schizophrenia.

## Data Availability

The datasets generated and/or analyzed during the current study are not publicly available due the terms of consent to which the participants agreed, but may be available from the corresponding author on reasonable request.

## Abbreviations

HSI: Heavy smoking index
SHEAPS: Study of health behavior in people with schizophrenia
